# Evaluating the impact of a community-based parent training programme for children with cerebral palsy in Ghana

**DOI:** 10.1371/journal.pone.0202096

**Published:** 2018-09-04

**Authors:** Maria Zuurmond, David O’Banion, Melissa Gladstone, Sandra Carsamar, Marko Kerac, Marjolein Baltussen, Cally J. Tann, Gifty Gyamah Nyante, Sarah Polack

**Affiliations:** 1 International Centre for Evidence in Disability, London School of Hygiene and Tropical Medicine, London, United Kingdom; 2 School of Medicine, Emory University, Atlanta, Georgia, United States of America; 3 Institute of Translational Medicine, University of Liverpool, Liverpool, United Kingdom; 4 Physiotherapy Department, Korle Bu Teaching Hospital, Accra, Ghana; 5 Department of Population Health, London School of Hygiene and Tropical Medicine, London, United Kingdom; 6 CBM West Africa Regional Office, Accra, Ghana; 7 Department of Infectious Disease Epidemiology, London School of Hygiene and Tropical Medicine, London, United Kingdom; 8 Department of Applied Health, University of Ghana, Accra, Ghana; IRCCS E. Medea, ITALY

## Abstract

**Background:**

In low and middle-income settings, where access to support and rehabilitation services for children with disabilities are often lacking, the evidence base for community initiatives is limited. This study aimed to explore the impact of a community-based training programme for caregivers of children with cerebral palsy in Ghana.

**Methods:**

A pre and post evaluation of an 11-month participatory training programme (“Getting to Know Cerebral Palsy”) offered through a parent group model, was conducted. Eight community groups, consisting of a total of 75 caregivers and their children with cerebral palsy (aged 18 months-12 years), were enrolled from 8 districts across Ghana. Caregivers were interviewed at baseline, and again at 2 months after the completion of the programme, to assess: quality of life (PedsQL^™^ Family Impact Module); knowledge about their child’s condition; child health indicators; feeding practices. Severity of cerebral palsy, reported illness, and anthropometric measurements were also assessed.

**Results:**

Of the child-caregiver pairs, 64 (84%) were included in final analysis. There were significant improvements in caregiver quality of life score (QoL) (median total QoL 12.5 at baseline to 51.4 at endline, P<0.001). Caregivers reported significant improvements in knowledge and confidence in caring for their child (p<0.001), in some aspects of child feeding practices (p<0.001) and in their child’s physical and emotional heath (p< 0.001). Actual frequency of reported serious illness over 12-months remained high (67%) among children, however, a small reduction in recent illness episodes (past 2 weeks) was seen (64% to 50% p < 0.05). Malnutrition was common at both time points; 63% and 65% of children were classified as underweight at baseline and endline respectively (p = 0.5).

**Conclusion:**

Children with cerebral palsy have complex care and support needs which in low and middle-income settings need to be met by their family. This study demonstrates that a participatory training, delivered through the establishment of a local support group, with an emphasis on caregiver empowerment, resulted in improved caregiver QoL. Despite less effect on effect on child health and no clear effect on nutritional status, this alone is an important outcome. Whilst further development of these programmes would be helpful, and is underway, there is clear need for wider scale-up of an intervention which provides support to families.

## Introduction

There are an estimated 150 million children globally living with a disability [1]. The vast majority (80%) of these children live in resource poor settings, where the rehabilitative services available are extremely limited [2]. Cerebral palsy, is the most common cause of physical impairment in children [3]: the prevalence is estimated to be 2–3.5 cases per 1000 live births [4] but is likely to be higher in low and middle-income countries (LMIC) [5]. Children with cerebral palsy often have multiple co-morbidities—including visual, hearing, and intellectual impairment, and epilepsy—requiring a multi-disciplinary approach to care and support over their lifetime [4]. They are more likely to experience poor health [2, 6] and have diverse rehabilitation needs, yet they often face a range of barriers in accessing health and education services [7, 8]. This contravenes the rights of children with disabilities to health care, education and social participation enshrined in the UN Convention on the Rights of Persons with Disabilities [9]. Further, the Sustainable Development Goals emphasise the importance of ‘leaving no one behind’ [10] and the Global Strategy for Women Children and Adolescent’s health [11] demands a more transformative agenda, moving beyond just survival, for maternal child and adolescent health.

Evidence shows that caregivers of children with disabilities in low resource settings are more likely to experience stress, social isolation, emotional and physical impacts [12–15]. The scarcity or complete absence of rehabilitation services in LMICs can result in families providing the bulk of care for their child, often with little or no access to training and support. Specifically in the Ghana context, medical rehabilitation services have been described as being minimal [3, 16], with only an estimated 150 registered physiotherapists for a population of 28.2 million, and the first cohort of nationally trained occupational therapists only graduating in 2017 [17]. While there have been calls for community and home-based programmes to improve care and support, emphasising the central role of families [18], these are currently lacking, and there is a dearth of studies evaluating their impact on children and their caregivers [19–21].

**Fig 1:**
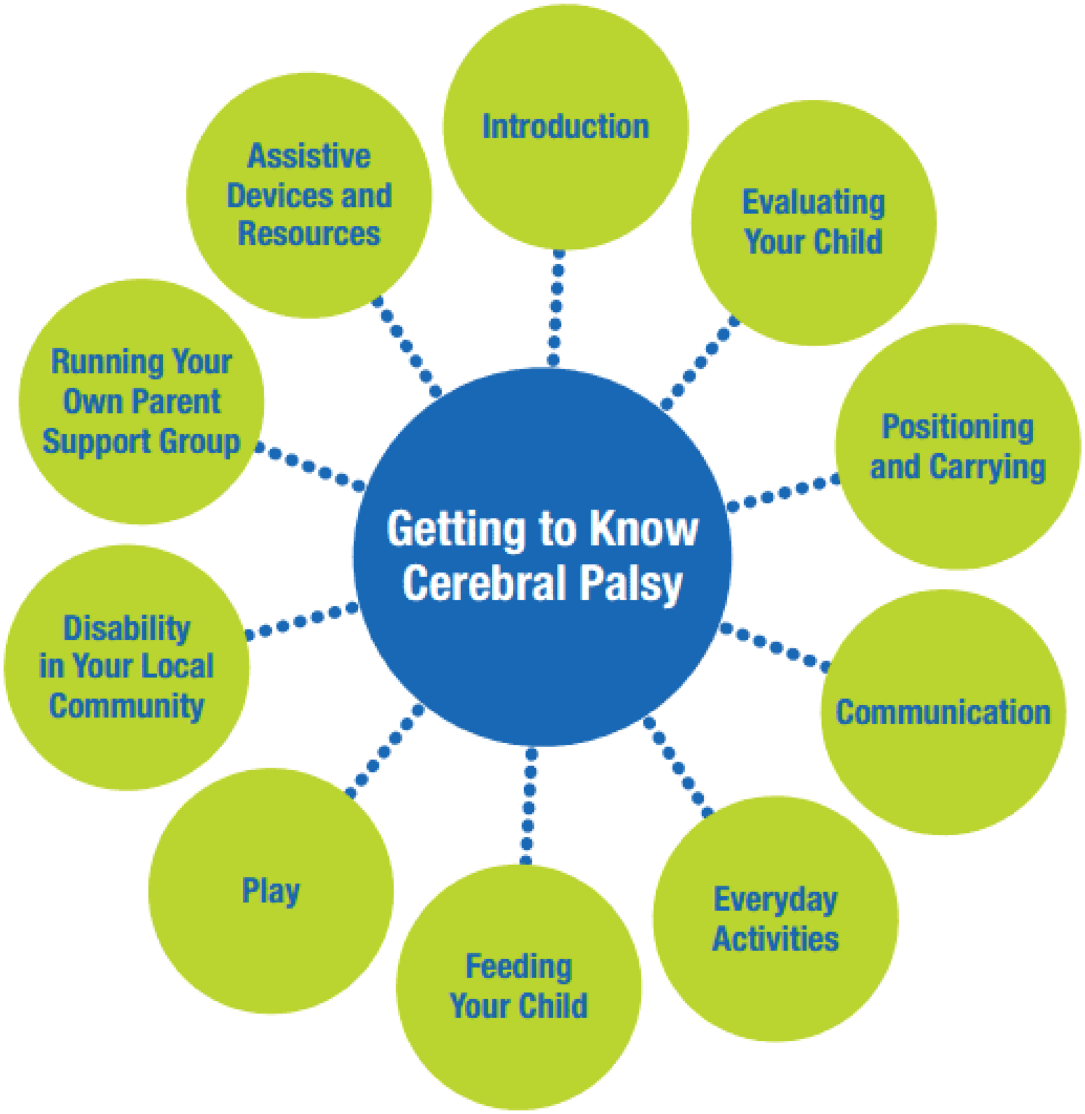
Modules of training programme.

In response to this recognised need for home and community-based interventions, the International Centre for Evidence in Disability (ICED) developed a participatory caregiver training package for children with cerebral palsy ‘*Getting to know cerebral palsy’ (GTKCP)* [22], adapted from a training resource called Hambisela [23]. GTKCP aims to empower caregivers and to improve care and support for children, within a rights-based framework. Using a participatory approach, a local parent support group was established, and they received monthly training sessions, consisting of 10 modules which are run over 11–12 sessions (Fig 1). The programme was developed through action research in Bangladesh [22] and has since been widely implemented across a variety of LMIC settings. However, empirical evidence is lacking on the impact of this programme on the children with cerebral palsy or their caregivers. Exploring this is important for identifying and informing improvements to the programme.

The aim of this study was to evaluate the impact of the GTKCP parent training programme on children with cerebral palsy and their caregivers in Ghana. Specifically, we aimed to explore the impact on caregiver quality of life and knowledge and attitudes towards caring for the child, and on the child’s health and nutritional status.

## Methodology

This study was a pre/post mixed-methods impact evaluation of the GTKCP parent training programme in Ghana. This article reports on the quantitative component of the study.

### Project location and study participants

Participants were recruited from across eight districts and four regions (Upper East, Greater Accra, Brong Ahafo, Ashanti) in Ghana. Sites were selected by the project implementation partner (The Presbyterian Church of Ghana) because of their existing primary health care or community-based programmes, which allowed for onward referral of children in the study to other services, such as accessing assistive devices. A total of 75 children and their caregivers were newly recruited through existing community screening programmes for cerebral palsy, hospital physiotherapy records of children diagnosed with cerebral palsy in the last six months, and from additional community screening in one site.

Cerebral palsy was diagnosed by a qualified clinician (developmental paediatrician or physiotherapist). Eligibility criteria were: a confirmed diagnosis of cerebral palsy (of any type) and age 18 months-12 years. Children may have had some previous physiotherapy, but caregivers were excluded if they were already a member of a parent support group.

### Intervention: Parent training programme

Caregivers were invited to a newly established local support group and offered a total of eleven once-a-month group training sessions each approximately three hours, in a nearby community setting. There were 8–10 parents per group. Although sessions were led by the facilitators, there was an emphasis on problem solving and peer support, underpinned by adult learning theory and an emphasis on participatory approaches to facilitate empowerment of the caregivers. Support groups were maintained following this training programme intervention, led by expert mothers, but this evaluation was conducted on completion of the initial training input following establishment of the groups.

The modular topics covered a range of issues as shown in Fig 1. In addition, all families were visited once per month by one/or both facilitators, for an average of 45 minutes, in order to provide one to one follow up support to the primary caregiver, and to discuss the training material with other family members.

The GTKCP programme was delivered by a pair of facilitators: a physiotherapist or physiotherapist assistant together with one primary health worker. A total of 13 facilitators underwent a one-week training from a master trainer and delivered to eight groups.

The fidelity of the training approach was monitored using the following: one face to face visit to at least one training session, and a monthly phone call to each group facilitator by the project coordinator and/or an experienced physiotherapist. A ‘Whats App’ group was set up and used by facilitators for sharing their implementation experiences and enabled some monitoring of quality. Although intended as a community-based programme, in some of the more rural areas, the families were quite dispersed, and some had to travel up to one hour to attend the group sessions. Transport costs were covered by the project.

### Data collection

**Fig 2:**
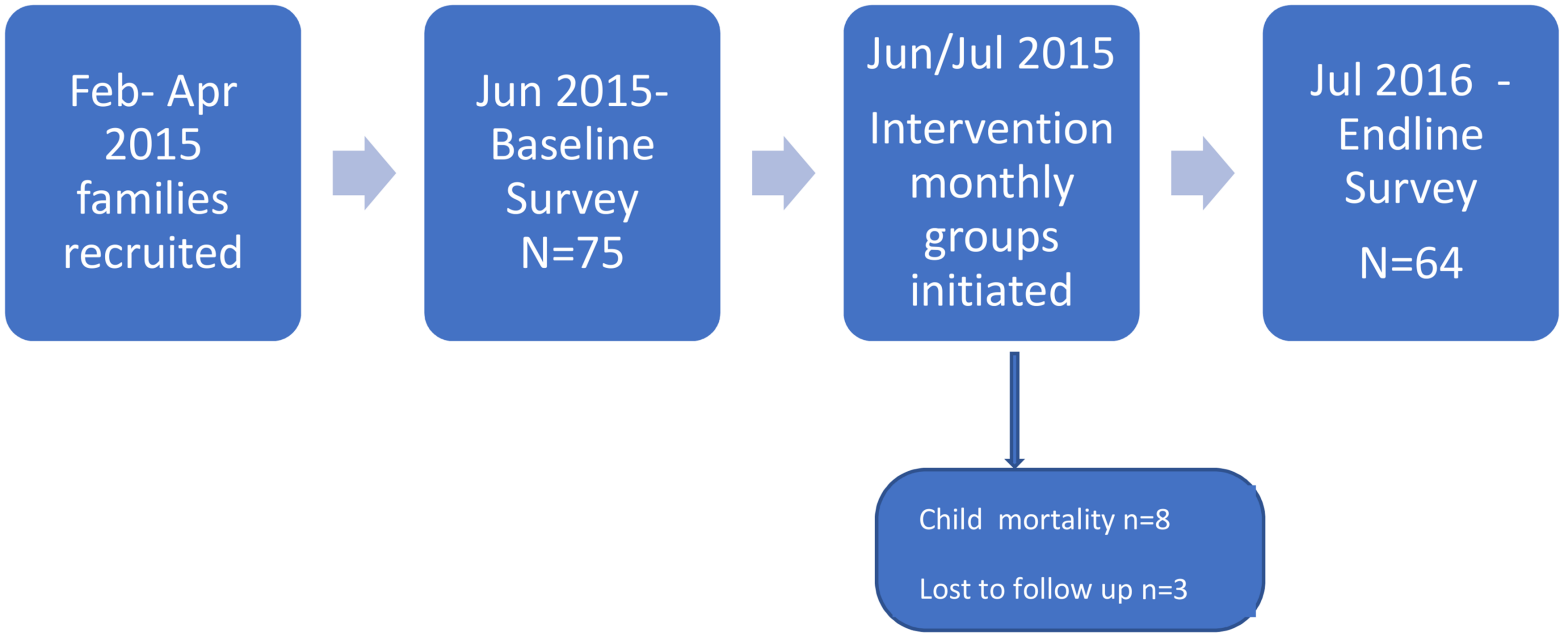
Pre/Post evaluation design pathway.

Primary caregivers were interviewed at home at least one month prior to the intervention (baseline) using a structured questionnaire in June 2015. Caregiver data collected included: socio-demographics, quality of life and knowledge in caring for their child. Data on the child included demographics, severity of cerebral palsy, health status, anthropometry and reported feeding difficulties. Data were collected electronically using a tablet (Google Nexus 7) and Open data kit software. The same respondent (primary caregiver) was re-interviewed 13 months later, 1–2 months after completion of the last training session (endline). See Fig 2

Amongst caregivers, the PedsQL^™^ Family Impact measure [24], was used to quantitatively assess the impact of paediatric conditions on the quality of life of parents and the family. The PedsQL^™^ measures parents’ self-reported physical, emotional, social, and cognitive functioning, communication, and worry, as well as daily activities of the family, and family relationships, and has been validated and tested in different international settings [25–27]. Linguistic validation of the PedsQL tools was conducted in three languages Twi, Gruni, and Kusaal) which included forward and back translations by linguistic experts and further modification following cognitive field testing in the three sites, in line with the translation guidelines [28]. Caregivers were asked about their level of knowledge and confidence in caring for their child with five possible response options from ‘none’ to ‘lots’ of knowledge or confidence, and additional questions to assess their experience of treatment and diagnosis.

Severity of cerebral palsy was assessed using the Gross Motor Function Classification System (GMFCS) [29], an internationally-recognised system, which classifies cerebral palsy according to five levels of physical functioning. Data on the child’s feeding were collected using structured questions adapted, with input from the author, from a previous study of children with cerebral palsy in Bangladesh [30]. Eight questions were asked about frequency of difficulties with different aspects of eating and drinking, rated on a five-point scale from ‘never’ to ‘always [30]. Reported health of the child was measured with three questions taken from the Ghana DHS survey about whether their child has experienced diarrhoea, fever or cough in the last two weeks [31], and one question about experience of a serious health condition requiring hospital admission in the last 12 months.

Anthropometric measures were taken using standardised protocols for our population. Standing height was recorded to the nearest 0.1cm for children > 5 years. Recumbent lengths were taken for children <5 years, where possible, and knee height [32] (CLPR65 Anthropometric Caliper, MediForm, Oregon, USA) was recorded for all children to the nearest 0.1cm. Following WHO best-practice procedures [33], height and length measurements were obtained twice by two independent observers: the average was taken as the final measurement if they agreed to within <0.5cm, else both re-measured until agreement was achieved. Mid Upper Arm Circumference (MUAC) was recorded for all children (mm gradation MUAC tape, Teaching Aids at Low Cost. Weight was recorded to the nearest 0.1kg. Children unable to stand were held by their caregiver, then the caregiver was weighed separately to calculate the child’s weight.

### Data analysis

Data were analysed using Stata 13 [34]. Total and summary scores from the PedsQL were calculated as the sum of all items divided by the number of items answered. These scores were converted into scores out of 100 with higher scores indicating better quality of life [24]. For the purposes of analysis, the 5 level GMFCS was reclassified into three groups: mild (levels 1 and II), moderate (levels III) and severe (levels IV and V). A composite feeding score comprised of the eight feeding/drinking questions was generated which ranged from 0 (extreme difficulties) to 100 (no difficulties). To assess the internal consistency of this composite scale we calculated Cronbach’s alpha coefficients, all which were >0.7 with item-total correlations >0.3 as per recommended guidelines for reliability [35]. Knee height was used as a proxy measure where standing height could not be performed [32]. The line of best fit on a scatter graph of the relationship between recumbent length and knee height was used to predict the heights of those children for whom data were unobtainable. This method was used because of a lack of validated published formula (for converting knee height to height) available for this specific study population. Z scores were calculated based on WHO growth standards using the Emergency Nutrition Assessment and WHO Anthroplus software [36] for weight-for-age (WAZ) (children <10years only), height-for-age (HAZ) and weight-for-height (WHZ) (children ≤5 years only) Children with WAZ/HAZ//WHZ scores between <2 and ≥3 were defined as stunted/wasted/underweight respectively and those with z scores <-3 were defined as severely. For MUAC, wasted was defined as 115mm to 124mm and severe wasting as <115mm. Children with extreme z-score values (greater than >5 or 6/less than -5 or -6, depending on the measure) were excluded from the analysis since they were more likely measurement errors than truly very large or small [37]. The prevalence of stunting, wasting and underweight was calculated, as well as the mean z-scores.

The standard mortality ratio was calculated with data from standard UN child model life tables for children 1–5 years [38] and those from Ghana for children of 1–12 years [39].

Outcomes at baseline and endline were compared using the McNemars tests for binary variables. Continuous variables were compared using the paired t-test (normally distributed data) or Wilcoxon sign rank test (if data were skewed). Analysis were restricted to respondents who were included at both baseline and endline.

### Ethics

Ethics approval was obtained from the London School of Hygiene and Tropical Medicine and from the Noguchi Memorial Institute for Medical Research (NMIMR), University of Ghana. Informed written consent was obtained from all participating caregivers, with a signature or thumbprint as appropriate. Children were referred to nutritional services and for assistive devices according to clinical need, and the CBM (international NGO) child protection policy was adhered to.

## Results

### Training programme

The adherence by facilitators to the training approach was overall assessed to be ‘good’ by the project coordinator, who visited each group once during the project period. Attendance at the training sessions was high with 92% of caregivers completing all training sessions.

### Study population

A total of 75 primary caregiver-child pairs were included at baseline and 64 (84%) and endline. Of those lost to follow up, eight died, and three did not complete the training programme (attended <3 sessions). Socio-demographic characteristics between study participants and those lost to follow-up were similar except girls were more likely to be lost than boys (p = 0.03) (see Table 1).

**Table 1:** Study population characteristics.

	n	%
**Child variables**		
**Sex**		
Male	38	59
Female	26	41
**Child age Group**		
18months-2 years	25	39
3–4 years	21	33
5+ years	18	28
**Cerebral Palsy severity**		
Mild	15	23
Moderate	17	27
Severe	32	50
**Caregiver variables**		
**Age group**		
<30	19	30
30–40	29	45
>40	16	25
**Sex**		
Male	1	2
Female	63	98
**Relationship to child**		
Mother	51	80
Father	1	2
Grandparent	10	16
Sibling	2	3
**Worked in past month (other than domestic)**	27	42
**Attended school**	28	44
**Highest level of schooling completed**		
None	33	43
Primary	18	24
Secondary	22	29
Tertiary	3	4
**Marital status**		
Married/living together	43	67
Divorced/separated	6	9
Widowed	5	8
Never married/lived together	10	16
Where does child’s biologic father live*		
Not in household	38	51
In household	35	47
Not known	2	3
**How often has father seen child in past 6 months**		
Daily	37	50
Monthly	3	4
< monthly	11	16
Not seen	20	27
Not known	2	3
**Occupation**		
Farming	24	32
Trader	20	27
Small business (e.g. dress making, hairdressing)	22	30
Professional	5	7
Other	3	4

*Included fathers who had died (n = 2)

Table 1 describes the characteristics of the study population. The majority, (72%) of children were under 5 years with a mean age of 3.8 years (SD 2.69). Half of the children had severe cerebral palsy. Caregivers were mainly (98%) female and the majority were mothers (80%) or grandparents (16%); 67% were married, whilst only half of the biological fathers lived in the same house as their child and 43% of the children saw their father less than monthly. 89% described their profession as farming, trading, or a small business such as tailoring. Although, only 42% said they had been able to work in the last month. See Table 1 for details.

### Impact of the programme on the caregiver

The average total caregiver QoL score was low (12.5, SD 18.6) at baseline and increased significantly to 51.4 (SD 23.6) at follow up (p<0.001). The improvement was significant across all three summary scales (parent, family, and combined) and across eight domains of physical, emotional, social, cognitive, communication functioning, worry, daily relationships and family relationships (p<0.0001). We assessed this change among caregivers of children with mild, moderate, and severe cerebral palsy separately and found significant improvements in QoL was evident within each severity group (p <0.001)Table 2.

**Table 2:** Baseline and endline caregiver QoL scores and mean change between the two-time points.

	Baseline Median score IQR)	Endline Median score (IQR)	P-value*	Mean change (95% CI)
**Summary scores**				
Total	12.5 (21.5)	51.4 (23.6)	P<0.001	+31.3 (25.6–37.0)
Parent	13.8 (21.3)	47.5 (25.6)	P<0.001	+28.9 (22.9–34.9)
Family	14.1 (46.9)	57.8 (30.0)	P<0.001	+31.9 (23.5–40.3)
**Sub-scale scores**				
Physical functioning	6.7 (27.1)	50.0 (50.0)	P<0.001	+26.7 (19.2–34.3)
Emotional functioning	10.0 (22.5)	47.5 (42.5)	P<0.001	+33.4 (26.1–40.8)
Social functioning	6.3 (28.1)	37.5 (56.3)	P<0.001	+25.9 (17.8–34.1)
Cognitive functioning	10.0 (35.0)	50.0 (40.0)	P<0.001	+29.2 (21.8–36.6)
Communication functioning	8.3 (16.7)	66.6 (37.5)	P<0.001	+45.4 (37.5–53.4)
Worry	10.0 (20.0)	50.0 (35.0)	P<0.001	+31.7 (24.4–39.1)
Daily activities	0 (16.7)	50 (45.8)	P<0.001	+32.3 (23.2–41.4)
Family relationships	5.0 (50.0)	62.5 (31.6)	P<0.001	+31.6 (21.1–42.2)

*Wilcoxon Sign Rank test comparing baseline and endline scores; NB: Baseline and endline data were skewed and therefore median scores and a non-parametric statistical test were used. Change scores were normally distributed and therefore mean values were derived. IQR = Interquartile Range

### Knowledge and confidence in taking care of child

At baseline, only 13% of caregivers had heard of the condition ‘cerebral palsy’. Overall levels of diagnosis of cerebral palsy prior to this study were very poor: 49% of caregivers claimed they had never received a diagnosis, 15% had been told by a health professional it was ‘something related to the brain’ and 21% reported a diagnosis provided by a traditional healer. At baseline, the vast majority (94%) reported no or low levels of knowledge of their child’s condition (Table 3). At endline, there was a significant increase in the proportion reporting good/lots of knowledge: from 6% to 73% (p<0.001). In terms of confidence in how to care for their child, at baseline half (49%) of caregivers reported a low level or no confidence. At endline, the proportion who felt they had good or lots of confidence had increased significantly from 36% to 89% (P<0.001). See Table 3.

**Table 3:** Caregiver knowledge and confidence and child’s health and wellbeing at baseline and endline.

	Baseline N (%)	Endline N (%)	P* value
**Caregiver knowledge in caring for child**			
None/low knowledge	60 (94%)	17 (27%)	<0.001
Good or lots of knowledge	4 (6%)	47 (73%)	
**Caregiver confidence in caring for child**			
None/low confidence	41(64%)	8 (13%)	<0.001
Good or lots of confidence	23 (36%)	89%)	
**Caregiver’s perception of child’s physical health**			
Poor/fair	42 (66%)	17 (27%)	<0.001
Good/very good/excellent	22 (34%)	47 (73%)	
**Caregiver’s perception of child’s emotional health**			
Poor/fair	41 (64%)	23 (36%)	<0.001
Good/very good/excellent	23 (35%)	41 (64%)	
**Child had serious health problem in past year**	45 (70%)	43 (67%)	0.63
**Child had illness in past 2 weeks:**			
Diarrhoea	8 (13%)	12 (18%)	0.31
Fever	24 (38%)	16 (25%)	0.13
Cough / Difficulty breathing	25 (39%)	16 (25%)	0.06
Any of above 3 illnesses	41 (64%)	32 (50%)	0.05

*P-value from McNemars test comparing baseline and endline

### Feeding and drinking

**Fig 3:**
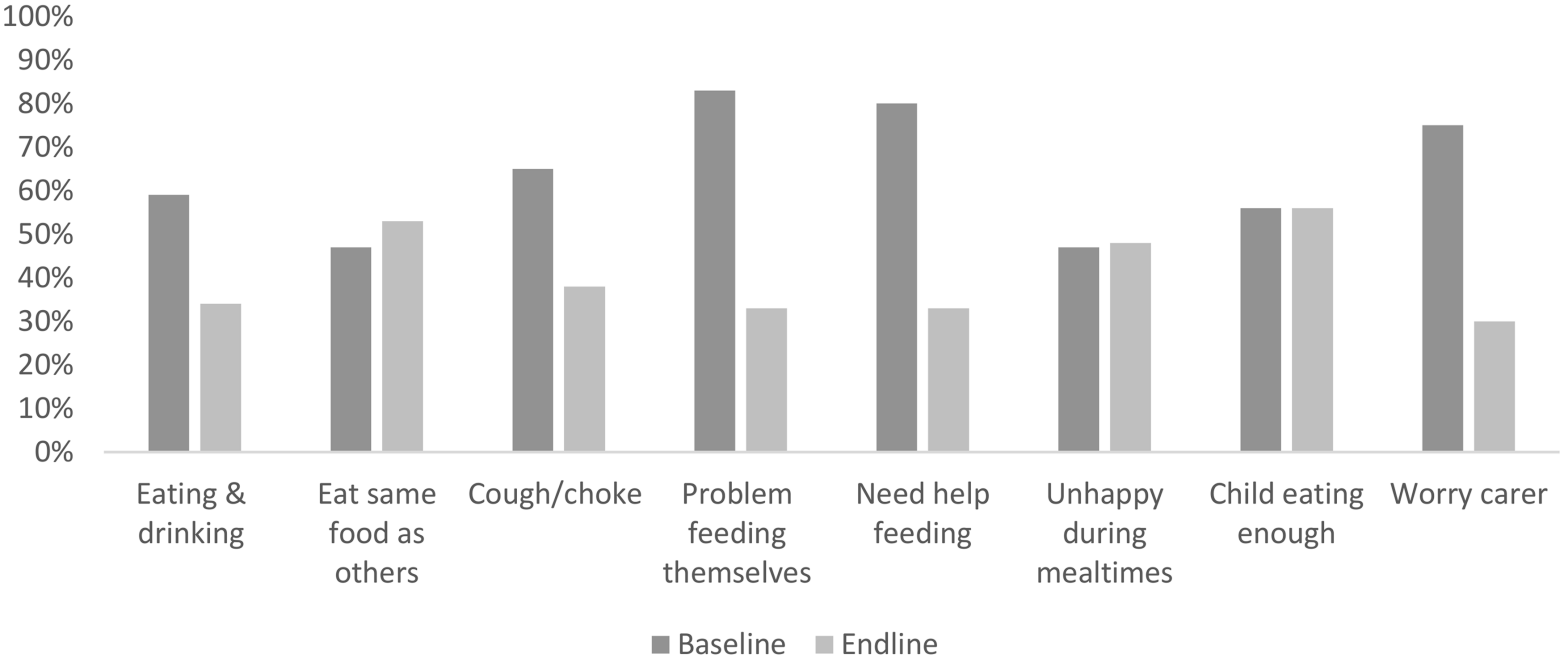
Proportion of caregivers reporting problems always/often with different aspects of child’s feeding at baseline and endline.

At baseline, at least 45% of caregivers reported problems often/always with the eight different domains of feeding and drinking as shown in Fig 3. At endline, there was a significant reduction in the proportion of parents who reported problems often/always in four domains (child eating and drinking, self-feeding, needs help feeding and caregiver worry about child feeding). Responses to the other domains (choking, eating the same as others, being unhappy during mealtimes, whether the child is eating enough) remained similar between baseline and follow up. In terms of the composite feeding score, there was a significant improvement in the median score from 29.7 (SD 31.4) at baseline to 51.6 (29.8) at endline (p<0.001).

### Child’s health and wellbeing

There were significant improvements in caregiver’s perception of their child’s physical and emotional health. The proportion of caregivers rating their child’s health as good, very good, or excellent increased from 34% (baseline) to 73% (endline, p<0.001) for physical health and from 36% to 64% for emotional health (p<0.001, Table 3). However, most children had experienced a serious health problem (i.e. a problem the caregiver felt required treatment) in the last 12 months at both baseline (70%) and endline (67%). Illness in the last two weeks was common with 64% of the population at baseline reporting illness in the past two weeks (at least one of: fever (38%), Cough/breathing difficulties (39%) diarrhoea (13%)). At endline, this was lower with 50% (p = 0.05) of caregivers reporting illness in the past two weeks: (fever: 25%, Cough/breathing difficulties: 25%; diarrhoea: 18%). See Table 3.

### Nutrition

As shown in Table 3, the prevalence of malnutrition was high at both time points. At baseline, 63% of children were classified as underweight and this remained similar at endline (p = 0.5). The proportion of children classified as stunted was 53.8% at baseline and 64.1% at endline (p = 0.06). Wasting was evident in 60% of children (< 4 years only) at baseline with 45.2% at endline (p = 0.01). Using MUAC, 15.2% were classified as wasted at baseline and 21.7% at endline.

We compared the proportion of children who were underweight at baseline and endline for children with mild, moderate, and severe CP separately and found the trends within each severity level were similar to that for the full population combined. See Table 4.

**Table 4:** Prevalence of malnutrition among children at baseline and endline.

	All ages**				Children < 5years (at baseline)			
	Baseline		Endline		Baseline		Endline	
	N	% (95% CI)	N	% (95% CI)	N	% (95% CI)	N	% (95% CI)
**Weight for Age**								
‘Normal’	21	36.8 *(25.1–50.3)	20	34.5 (23.1–48.8)	15	34.9 (21.7–50.7)	15	34.1 (21.3–49.7)
Underweight	14	24.5 (14.8–37.7)	10	17.2 (10.4–29.6)	12	27.9 (16.2–43.6)	9	20.5 (10.7–35.5)
Severely underweight	22	38.5 (28.5–52.6)	28	48.2 (35.4–61.3)	16	37.2 (23.7–53.0)	20	45.5 (31.0–60.7)
*Mean z-score (95% CI)*		-2.6 (-2.9- -2.2)		-2.8 (-3.1- -2.3)		-2.6 (-2.9- -2.1)		-2.7 (-3.2- -2.3)
p-value*				P = 0.08				P = 0.06
**Height for Age**								
‘Normal’	29	46.0 (33.9–58.6)	23	35.9 (24.9–48.7)	24	52.2 (37.4–66.6)	18	39.1 (25.8–54.3)
Stunted	17	26.9 (17.3–39.5)	16	25.0 (15.7–37.4)	10	21.7 (11.8–36.5)	12	26.1 (15.1–41.1)
Severely stunted	17	26.9 (17.3–39.5)	25	39.1 (27.7–51.8)	12	26.1 (15.1–41.1)	16	34.8 (22.1–50.0)
*Mean z-score (95% CI)*		-2.3 (-2.6- -1.9)		-2.7 (-3.0- -2.4)		-2.2 (-2.6- -1.8)		-2.6 (-3.0- -2.2)
p-value*				P = 0.003				P = 0.002
**Weight for height**								
‘Normal’					12	40.0 (23.4–59.1)	17	54.8 (36.5–71.9)
Wasted					12	40.0 (23.4–59.1)	4	12.9 (4.6–31.6)
Severely wasted					16	20.0 (8.8–39.2)	10	32.3 (17.7–51.4)
*Mean z-score (95% CI)*						-2.1 (-2.5- -1.6)		-1.9 (-2.5- -1.3)
p-value (z scores)*								P = 0.24
**Mid Upper Arm Circumference**								
‘Normal’ (> = 125mm)					43	84.7 (70.7–92.7))	36	78.3 (63.5–88.1)
Wasted (115-124mm)					10	15.2 (7.2–29.2)	6	13.0 (0.6–26.8)
Severely wasted (<115mm)					0	0	4	8.7 (3.1–21.7)
*Mean MUAC (95% CI)*						144.8 (139.3–150.4)		144.4 (137.9–150.8)
p-value (mean MUAC)*								P = 0.8

*P value comparing z scores at baseline and endline.

** Weight for age calculated for children <10 years as per guidelines.

### Child mortality

In total, eight children died over the 12-month study period; mainly in the early part of the programme, all with severe cerebral palsy, and classified as malnourished at baseline and referred to nutrition services. A confirmed diagnosis for death was not provided by caregivers except in one case where sepsis and acute malnutrition was diagnosed. The standard mortality ratio for children 1–5 years, showed that the study children were 14.61 times more likely to die than children in the standard population for developing countries (P < 0.05). For the wider age range of children (1–12 years) the standard mortality ratio was 5.88 (P < 0.05), meaning that with cerebral palsy of this age were 5.88 times likely to die than children in the standard Ghana population.

## Discussion

This study in Ghana found significant improvements in QoL among caregivers of children with cerebral palsy who participated in a parent training programme offered through the establishment of a support group in a nearby community. Significant improvements were also found in caregiver reported knowledge and confidence in caring for their child, and in some aspects of child feeding. Reported serious health conditions and levels of malnutrition in these children were high both before and after the intervention.

The initial low caregiver QoL scores reported at baseline in this study aligns with a previous study in Bangladesh using the same tool [27] and other studies in LMIC which have found high levels of anxiety, depression, stress and poorer quality of life among caregivers of children with disabilities [12, 14, 27, 40–42]. The few studies which have included caregiver outcomes for home and community-based interventions, have generally focussed on one or two measures of improvements in maternal knowledge and stress, and parent satisfaction with services [43] [20] [44–46]. In our study, the reported improvement in knowledge and confidence in caring for their child is encouraging; and arguably, more importantly, the emphasis on problem solving and caregiver empowerment, within a support group setting, has the potential to offer a more sustainable mechanism for improving long term outcomes for the child and family. There is a clear need for such an approach which also supports task-sharing for rehabilitation, given the limited number of therapist and rehabilitation services in the Ghana context. However, this intervention does not aim to replace specialist services, rather for referrals to be strengthened, and for empowered caregivers to be able to demand these services, within a rights-based framework. Follow- up research with the support groups is now required to understand how these groups have been developed and sustained.

Evidence of improvements in child outcomes was more mixed. Some aspects of mealtimes and feeding their child were improved. Other studies have similarly highlighted the particular challenges of feeding for children with disabilities, including children with cerebral palsy [30, 47–50], and our baseline study showed feeding difficulties were also strongly associated with poorer caregiver QoL and child malnutrition [51]. Yet few studies have evaluated interventions aimed at improving nutritional outcomes for these children in low resource settings: we only found one study in Bangladesh study demonstrated improvements in nutritional outcomes through parental support and training, exclusively focussed on a feeding intervention [30].

Despite some positive changes in reported feeding experiences by the caregiver, levels of malnutrition remained high with some two-thirds of the population malnourished at both baseline and endline. This high level of malnutrition is mirrored in a study in Uganda [50] and is considerably higher than 2014 DHS estimates for the general population in Ghana where 11% of children under 5 years were underweight, 5% wasted and 19% stunted [31]. To some extent this reflects a vulnerable group whose growth potential—even in ideal environments—is below that of non-disabled children [52, 53]. However, unreached potential for growth clearly deserves further future attention. Whilst the training programme covers a range of topics, it is possible that: more emphasis on feeding and drinking practices would have improved anthropometry; more specific guidance on nutrient-dense feeds is required; further strengthening referrals pathways to nutrition programmes is needed. In addition, more attention should be given to the specific needs of younger children as they are weaned and transition to solids. There are also other documented complex challenges for inclusive child nutrition programmes including: the role that stigma and discrimination and poverty can play, the lack of inclusive nutritional services, and a need for stronger linkages in programming between nutrition and disability, poverty, and human rights [54].

The high mortality ratio highlights the extreme vulnerability of children of all ages, particularly for children aged 1–5 years who were almost 15 times likely to die than general child population of the same age. We were unable to identify other data on SMR among children with CP in LMIC. However, high rates of mortality have been noted in Bangladesh where 18% of 92 children with cerebral palsy died over a 3-year period; the most severely malnourished and severely disabled were most likely to die [55]. These findings underline the need to prioritise early intervention to prevent and manage serious malnourishment, and to establish closer linkages between disability and malnutrition.

In this study the child’s emotional and physical health was reported as improved by the caregiver but reported difficulties with breathing (25%) and diarrhoea (18%) were still very high in comparison with the national Ghana DHSS survey data of 12% of children that experience diarrhoea, and 4% with ARI, at 2 weeks previous to the survey [31]. Our findings are aligned with the small number of studies which demonstrate children with disabilities are more likely to experience poorer health [2, 6, 56, 57].

The absence of fathers in households of children with cerebral palsy highlighted in this study deserves attention. Overall there is little data from LMICs exploring this issue, despite the multi-fold implications, including impact on household income, on caregiving roles, the implications for any community and home -based intervention programme, and to ensure that any intervention does not further burden the gendered nature of caregiving. A detailed analysis of the intersectionality of gender and disability and caregiving is likely to be important to further strengthen this intervention.

Finally, the results show that a relatively simple intervention can improve outcomes for caregivers of children. There needs to be aspects of the training and approach which need strengthening, and this is underway. The model of establishing a support group, emphasising caregiver empowerment, potentially offers a more sustainable mechanism to address needs for a child with a disability who requires care over a lifetime. A longer-term re-assessment of caregivers and their children is recommended, also to better understand the sustainability of these support groups.

### Study strengths and limitations

Ours is one of very few studies evaluating the impact of a community based, family focussed intervention for children with cerebral palsy in low income settings. It was conducted across eight different sites in Ghana, using standardised questionnaires, and clinical assessment of all participating children.

The participant numbers were not large, they were comparable to other studies conducted with children with cerebral palsy [20, 43, 44]. The target families were identified in areas supported by the Presbyterian Church of Ghana, who typically work in areas of greater deprivation, and so there may have been a selection bias, with our sample more likely to be poorer than the general population. This may limit the generalisability of the findings and might also mean that impact was more limited by poverty.

We did not include a control group of caregivers and children who did not receive the intervention against which to compare, because of ethical reasons. We cannot rule out the role of social desirability in the responses of caregivers in their rating of QoL, caring and ratings of child health. We did not assess children’s quality of life, due to the lack of suitable tools for younger children, without using parents to offer a proxy measure. This should be explored in future studies. Future studies should also explore longer term impact of the training and sustainability of the support groups.

## Conclusion

There is a need for effective home and community-based programmes for children with disabilities, and their families. This study showed promising outcomes for improving the lives of the primary caregiver, following a relatively simple participatory training programme delivered through local support groups. With a focus on caregiver empowerment within a rights- based approach, the benefits are potentially more sustainable, with the right kind of ongoing support, and transformative for families. The results for the children were mixed and highlight an urgent need for more attention to be paid to nutrition and feeding, which are underway.
